# Temporal evolution of brain cancer incidence in the municipalities of Navarre and the Basque Country, Spain

**DOI:** 10.1186/s12889-015-2354-5

**Published:** 2015-10-05

**Authors:** María Dolores Ugarte, Aritz Adin, Tomás Goicoa, Itziar Casado, Eva Ardanaz, Nerea Larrañaga

**Affiliations:** Department of Statistics and O.R., Public University of Navarre, Campus de Arrosadía, Pamplona, 31006 Spain; Institute for Advanced Materials (INAMAT), Public University of Navarre, Campus de Arrosadía, Pamplona, 31006 Spain; Research Network on Health Services in Chronic Diseases (REDISSEC), Madrid, Spain; Navarre Public Health Institute, Calle Leyre 15, Pamplona, 31006 Spain; CIBER of Epidemiology an Public Health CIBERESP, Madrid, Spain; Public Health Division of Gipuzkoa, BIODonostia Research Institute, Government of the Basque Country, Nafarroa hiribidea 4, Donostia-San Sebastián, 20013 Spain

**Keywords:** Brain cancer incidence, Small areas, Space-time models, Relative risks

## Abstract

**Background:**

Brain cancer incidence rates in Spain are below the European’s average. However, there are two regions in the north of the country, Navarre and the Basque Country, ranked among the European regions with the highest incidence rates for both males and females. Our objective here was two-fold. Firstly, to describe the temporal evolution of the geographical pattern of brain cancer incidence in Navarre and the Basque Country, and secondly, to look for specific high risk areas (municipalities) within these two regions in the study period (1986–2008).

**Methods:**

A mixed Poisson model with two levels of spatial effects is used. The model also included two levels of spatial effects (municipalities and local health areas). Model fitting was carried out using penalized quasi-likelihood. High risk regions were detected using upper one-sided confidence intervals.

**Results:**

Results revealed a group of high risk areas surrounding Pamplona, the capital city of Navarre, and a few municipalities with significant high risks in the northern part of the region, specifically in the border between Navarre and the Basque Country (Gipuzkoa). The global temporal trend was found to be increasing. Differences were also observed among specific risk evolutions in certain municipalities.

**Conclusions:**

Brain cancer incidence in Navarre and the Basque Country (Spain) is still increasing with time. The number of high risk areas within those two regions is also increasing. Our study highlights the need of continuous surveillance of this cancer in the areas of high risk. However, due to the low percentage of cases explained by the known risk factors, primary prevention should be applied as a general recommendation in these populations.

## Background

Several studies have pointed out that the Autonomous Regions of Navarre and Basque Country show higher brain cancer incidence [1] and mortality [2, 3] risks than the rest of the Spanish regions. According to the latest estimated data published by the GLOBOCAN project, in 2012 a total of 2,056 and 1,661 new brain cancer cases were diagnosed in Spanish male and female population respectively, representing 6.1 and 4.2 cases per 100,000 inhabitants (age-standardized rates adjusted to world population) [4]. Although these rates are below the European average (6.3 and 4.6 in males and females respectively), data from the International Agency for Research on Cancer (IARC) showed that during the period 2003–2007, brain cancer incidence rates in Navarre and Basque Country provinces were among the highest rates registered for all regions and both genders [5]. With the aim of determining the possible causes that explain these high risk clusters in Spain, an exhaustive geographical study of the potential influence of land use variables was assessed [6]. No evidence on the possible association between the specific type of crop or land use and the distribution of the disease was found. Recently, a study of brain and central nervous system (CNS) cancer incidence in Navarre was performed to describe temporal trends of cancer incidence rates during the period 1973–2008 [7]. In that work, the area was considered as an explanatory variable dividing the observed cases into urban and non-urban areas. However, the geographical distribution of the disease within Navarre was not analyzed. A study of spatial mortality patterns for several cancer locations (stomach, colorectal, lung, breast, prostate and urinary bladder cancer) was also recently performed on 8,073 Spanish municipalities during the period 1989–2008 [8], but brain cancer was not studied. In this paper our interest lies in analyzing the temporal evolution of the geographical distribution of brain cancer incidence in the municipalities of Navarre and Basque Country. We are mainly interested in locating high risk municipalities within both Autonomous Regions. Health areas constituted by several municipalities were also considered as a new level of spatial aggregation to gain power in our analysis. According to the literature, just a small percentage of brain cancer cases can be explained by the only clearly established risk factors: genetic and environmental factors [9, 10], and ionizing radiations [11, 12]. This lack of clearly established factors is the reason why the analysis performed here becomes so crucial.

## Methods

### Ethics

This research has been performed with the approval of the ethics committee of the Public University of Navarre (code PI-004/14).

### Data source

The study is based on brain cancer incidence cases (International Classification of Diseases-10, code C71) recorded throughout the period 1986–2008 in Navarre and Basque Country population based cancer registries. The municipalities considered were those existing at the beginning of the period (year 1986). Later, there were some changes (some new municipalities arose) but then, the population of the new areas were aggregated to the municipality they belonged to in 1986, resulting in a total of 501 municipalities. The quinquennial population was provided by both the Statistical Institute of Navarre (IEN) and the Basque Country Statistical Institute (EUSTAT). Population for non census years was computed using linear interpolation. A total of 5,223 cases were recorded throughout the period 1986–2008 (1,214 in Navarre and 4,009 in the Basque Country), of which 2,891 were diagnosed in males and 2,332 in females. The expected cases per year and municipality ranges from 0 to 35.7, whereas the number of observed cases varies from 0 to 44. The overall incidence rate is about 9.6 per 100,000 inhabitants in Navarre and and 8.3 cases per 100,000 inhabitants in the Basque Country. Population sizes of the small areas (municipalities) considered in our study are highly unbalanced, where average population during the period 1986–2008 vary from 21 to 360,623. Mean values of the most populated municipalities of each health area are shown in Table 1.

**Table 1:** Mean values of the most populated municipalities of each health area in the period 1986–2008

Health area	Municipality	Population
Bilbao	Bilbao	360,623
Araba	Vitoria	214,687
Pamplona	Pamplona	181,683
Gipuzkoa	San Sebastián	179,814
Ezkerraldea-Enkarterri	Barakaldo	103,645
Uribe	Getxo	80,924
Bidasoa	Irún	56,044
Interior	Basauri	47,416
Bajo Deba	Eibar	30,183
Tudela	Tudela	28,465
Alto Deba	Mondragón	24,108
Goierri-Alto Urola	Azpeitia	13,581
Estella	Estella	12,958

### Statistical analysis

The statistical model used to smooth (relative) incidence risks is a mixed Poisson model including spatial and temporal correlation. It is explained in some detail in what follows. As mentioned above, Navarre and Basque Country (two Autonomous Regions in Spain) are divided into *n*=501 small areas (municipalities) labelled as *i*=1,…,*n* and data are available for time periods *t*=1,…,*T*. Let *E*_*it*_ represent the number of expected cases in region *i* and year *t* computed using age and sex-standardization with the population of the study as reference. The indirect standardization method allowed to compare each municipality in a certain year with the overall area throughout the entire study period. Then, conditional on the relative risks *r*_*it*_, the number of cancer incidence counts *O*_*it*_ is assumed to be Poisson distributed with mean *μ*_*it*_=*E*_*it*_*r*_*it*_. That is,

$$\begin{array}{@{}rcl@{}} \begin{array}{rcl} O_{it}|r_{it} & \sim & Poisson(\mu_{it}=E_{it}r_{it})\\ \log \mu_{it} & = & \log E_{it} + \log r_{it} \end{array} \end{array} $$

**Fig. 1:**
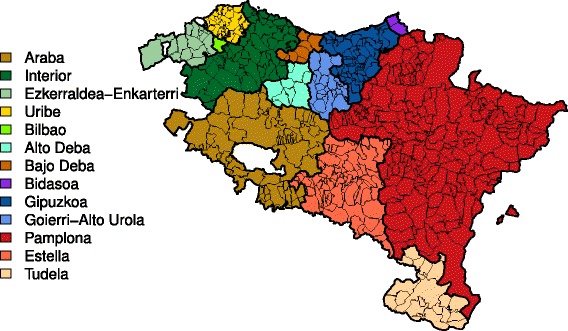
Map of the *n*=501 municipalities of Navarre and Basque Country aggregated by health areas

Several models have been considered to smooth the log-risks. The most common models in spatio-temporal disease mapping are possibly the non-parametric conditional autoregressive (CAR) models described in [13], where different types of space-time interactions between the main spatial and temporal random effects are proposed. Here, we considered a two-level spatial structure where municipalities were aggregated into larger health areas (see Fig. 1). The model can be seen as an extension of the models proposed by Schrödle et al. [14]. Specifically, the log-risks are modeled as

(1) $$ \log r_{it} = \alpha+\xi_{i}+\psi_{j(i)}+\gamma_{t}+\delta_{j(i)t}  $$

where *j*(*i*) denotes the health area (*j*=1,…,13) municipality *i* belongs to, *α* quantifies the logarithm of the global risk, *ξ*_*i*_ and *ψ*_*j*(*i*)_ represent the two-level spatial effects (municipality and health area effects respectively), *γ*_*t*_ (*t*=1,…,23) denotes temporal effects, and *δ*_*j*(*i*)*t*_ are space-time interaction effects.

The municipality-level random effects *ξ*_*i*_ were modeled initially using the CAR model modification proposed by Leroux et al. [15]. However, all the spatial variability was structured and then, an intrinsic CAR model was finally fitted. An exchangeable distribution was considered for the health area random effect, $\psi \sim N\left (0,\sigma ^{2}_{\psi } I_{m}\right)$. The temporal effect *γ*_*t*_ was modeled using a second order random walk to borrow strength from second-order temporal neighbors. The interaction term *δ*_*j*(*i*)*t*_ designates specific and independent temporal trends for each health area (Type II interaction in [13]). The model was fitted from an empirical Bayes approach using the well known penalized quasi-likelihood (PQL) technique [16–18]. To avoid identifiability problems all the random effects of Eq. (1) were reparameterized using appropriate transformation matrices based on the eigenvector decomposition of the variance-covariance matrices of these random effects. Finally, spatial, temporal and spatio-temporal patterns defined by the estimated log-risks were analyzed separately. The corresponding variances were computed using the delta method. Upper one-sided confidence intervals were constructed for the spatial effects and the true risk values to find high risk municipalities. When the lower limit was greater than one, the region was classified as a high risk region. All the analysis were carried out using R 3.1.2 software [19].

## Results and conclusions

**Fig. 2:**
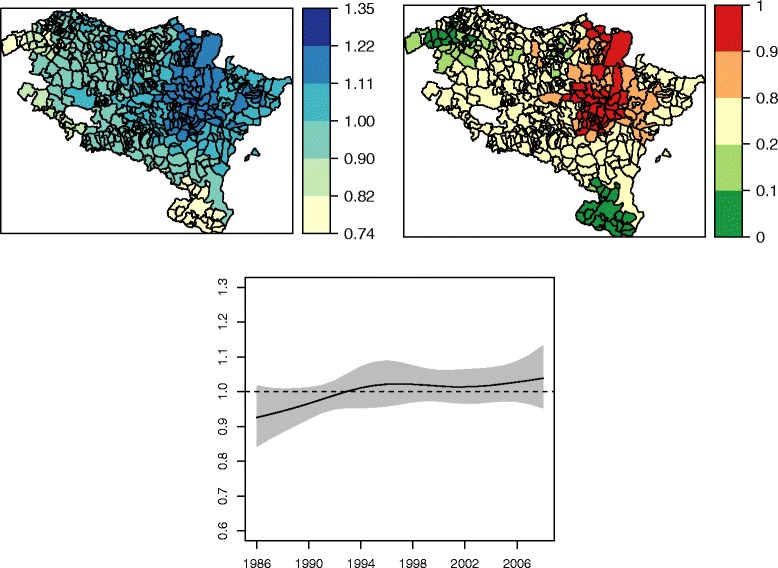
Geographical pattern of brain cancer incidence (*upper left* figure), significant high risk municipalities (*upper right*) and global temporal trend (*bottom*)

**Fig. 3:**
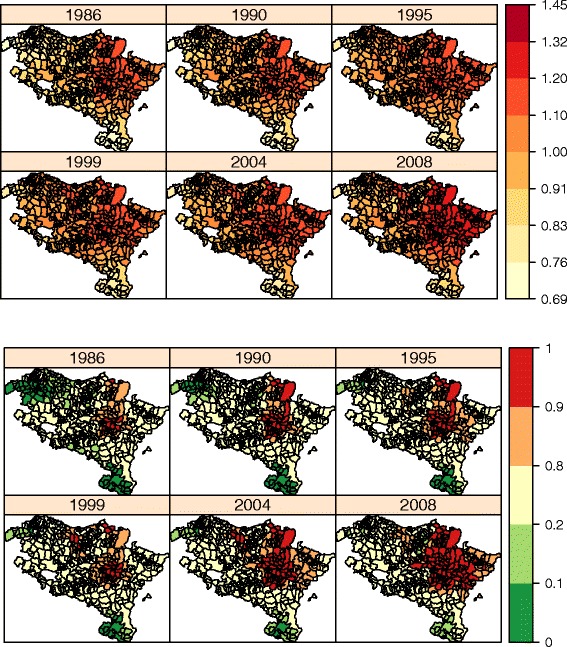
Temporal evolution of the geographical pattern of brain cancer incidence estimated risks (*up*) and corresponding maps of significantly high risk municipalities (*down*) of Navarre and Basque Country

The spatial pattern explains most of the total variability of the relative risks (about 85 *%*), while the rest of variability is explained by the temporal (9 *%*) and spatio-temporal (6 *%*) patterns. Figure 2 on the upper-left shows the spatial incidence risk pattern associated to each municipality and constant along the whole period, while high risk municipalities are displayed on the upper-right hand in Fig. 2. The areas with the highest spatial risks are located mainly in a region surrounding the capital of Navarre, Pamplona, but there are also a few municipalities with significant high risks in the northern part of the region, specifically in the border between Navarre and the Basque Country (Gipuzkoa). Conversely, the areas with the lowest spatial risks belong to the health area of Tudela (South of Navarre) and Ezkerraldea-Enkarterri (West of the Basque Country). The temporal risk pattern common to all regions is displayed at the bottom of Fig. 2. An increasing risk is observed from 1986 till approximately 1994, where the risk remains stable until 2002. From there on an upward trend is observed. This global increase in brain cancer incidence risk is also seen in Fig. 3, where the temporal evolution of the geographical incidence pattern is represented for some years of the period 1986–2008.

**Fig. 4:**
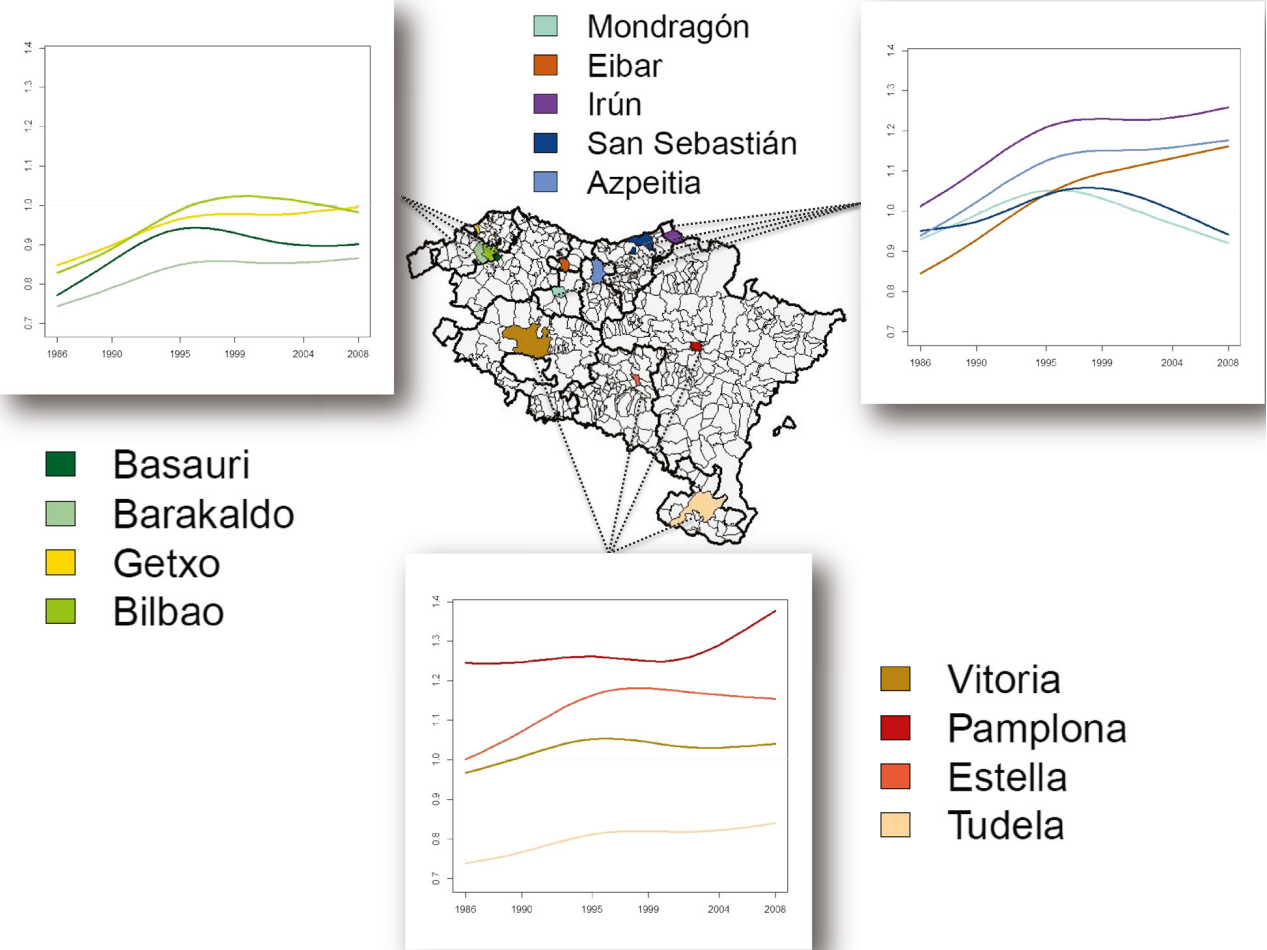
Temporal evolution of the estimated brain cancer incidence risks $\hat {r}_{\textit {it}}$ for the more populated municipalities of each health area in Navarre and the Basque Country

Finally, temporal evolutions of relative incidence risks are plotted in Fig. 4 for the most populated municipalities of each health area. In general, differences are observed among specific risk evolutions. For instance, the areas of Bilbao, Mondragón and San Sebastián show a decrease in their relative incidence risks during the second half of the period 1986–2008, while other municipalities like Azpeitia, Eibar, Irún and Pamplona in particular show an increase during the last years. These deviations from the global trend (see Fig. 2) are possible due to the flexibility of the selected model.

## Discussion

The model used in the data analysis is very rich, allowing to evaluate the evolution of the geographical pattern of brain cancer incidence in Navarre and the Basque Country. It has the potential to obtain altogether in one the geographical pattern for the whole period, the global temporal trend, and the temporal evolution of the geographical pattern. Previous models analyzing brain cancer incidence data in Navarre and the Basque Country only provided geographical [6] or temporal patterns [7]. Due to the large number of areas involved in our analysis and the evident differences in the risk evolution in each of them, the use of spatio-temporal models including space-time interactions becomes essential. Our analysis reveals that the number of high risk municipalities is increasing with time, and that the risk evolution for particular municipalities differs from the global temporal pattern. The model used is a non-parametric CAR model where different precision matrices can be used to model the spatio-temporal interaction term [13]. In our case, after considering alternative models to smooth the log-risks, a two-level (municipalities within health areas) spatial structure model was selected. The model also includes a random walk of order two for the temporal effects that borrows strength from second order time neighbors, and a temporally structured health area level interaction effect. Model fitting was carried out from a frequentist perspective and it was carefully programmed to take into account identifiability issues. Variability measures were also derived as they are not directly obtained from the fitting.

The brain cancer incidence data used in our analysis have been obtained from two internationally standardized population based cancer registries subject to quality controls. Nowadays population based cancer registries include benign brain tumors for a better understanding of brain cancer epidemiology. However, we have not included them in this study because we did not have this information available for the whole period 1986–2008. The last figures for different cancer registries published by the International Agency for Research on Cancer (IARC) [5] for the period 2003–2007, showed that CNS tumors including brain cancer (ICD-10 codes C70-72) incidence rates adjusted to world population were 7.8 and 7.2 for males, and 5.7 and 5.2 for females in Navarre and Basque Country respectively. Although these rates are high, there are other European regions with higher rates in both genders like Croatia, Norway, Serbia, Sweden and some regions of Italy and Poland. In Spain, rates ranged from 4.8 in Cuenca to 7.8 in Navarre for males, while for females the range varied between 3.0 in La Rioja and 5.7 in Navarre. In our analysis, significantly high risk areas were found in Pamplona and surrounding areas and also in municipalities close to the border between Navarre and Gipuzkoa. Similar results were observed in López-Abente et al. [6], where only geographical patterns of brain cancer incidence data were analyzed during the period 1978–1992. These results are also similar to previously published works on brain cancer incidence [1, 7] and mortality [2]. The findings in Etxeberria et al. [7] showed a higher brain cancer incidence rates in urban (Pamplona) rather than in rural areas. Although many agricultural chemicals and pesticides used in rural areas were believed to be brain cancer risk factors [6], the last data stand out that their effects in Navarre were small since the rates in rural areas were lower than in urban regions. Our analysis showed an increase of the risk along the study period. Part of this increase could be explained by the improvement of diagnostic techniques allowing for a more specific diagnosis of this tumor.

The geographical differences worldwide have been attributed, at least in part, to the accessibility to health services in general and to the use of new technologies in particular. This makes it possible to have better information on the morphology of tumors, particularly in older age groups, but it may also contribute to the diagnosis of incidental neoplasms [20]. The small number of cases, the long latency period, and the variations in study designs and available information, make it difficult to draw conclusions on specific brain tumors and individual risk factors. Brain tumors are rare tumors and their only clearly established risk factors are hereditary syndromes, ionizing radiation and age [21]. A great number of environmental expositions have been studied in adults aiming at explaining the exposition frequency but results were inconsistent. For example some studies have revealed the association between parental and subject occupation and brain cancer [22–24] but other studies have found no association [25, 26]. In 2011, the International Agency for Research on Cancer (IARC) of the World Health Organization (WHO) classified radio frequency (RF) as “possible carcinogenic to humans - 2B” [27] but no definite conclusions have been shown yet. Finally, Provost et al. [28] found that agricultural workers with the highest level of exposure to pesticides were twice as likely to be diagnosed with brain cancer as those with no exposure. However, pesticides are used in vineyards, and the areas of wine production in our study (the Ribera district of Navarre and La Rioja in the Basque Country) did show a low brain cancer incidence risk. The high risk municipalities that we found in this study are not related to any particular type of farmer-land or industrial setting. The results of the analysis performed in this paper are not conclusive as they are of descriptive nature. However, the study points out the relevance of using population based cancer registries to identify high risk areas related to environmental exposures. These registries are extremely valuable tools for providing useful and reliable information on brain cancer and other central nervous system tumors incidence [29]. Our study highlights the need of continuous surveillance of this cancer in the areas of high risk. However, due to the low percentage of cases explained by the known risk factors, primary prevention should be applied as a general recommendation in these populations.
